# A qualitative study of DRG coding practice in hospitals under the Thai Universal Coverage Scheme

**DOI:** 10.1186/1472-6963-11-71

**Published:** 2011-04-08

**Authors:** Krit Pongpirul, Damian G Walker, Peter J Winch, Courtland Robinson

**Affiliations:** 1 Department of International Health, Johns Hopkins Bloomberg School of Public Health, Baltimore, MD, USA; 2 Department of Preventive and Social Medicine, Faculty of Medicine, Chulalongkorn University, Bangkok, Thailand

**Keywords:** Diagnosis Related Group, Hospital Coding Practice, DRG Creep

## Abstract

**Background:**

In the Thai Universal Coverage health insurance scheme, hospital providers are paid for their inpatient care using Diagnosis Related Group-based retrospective payment, for which quality of the diagnosis and procedure codes is crucial. However, there has been limited understandings on which health care professions are involved and how the diagnosis and procedure coding is actually done within hospital settings. The objective of this study is to detail hospital coding structure and process, and to describe the roles of key hospital staff, and other related internal dynamics in Thai hospitals that affect quality of data submitted for inpatient care reimbursement.

**Methods:**

Research involved qualitative semi-structured interview with 43 participants at 10 hospitals chosen to represent a range of hospital sizes (small/medium/large), location (urban/rural), and type (public/private).

**Results:**

Hospital Coding Practice has structural and process components. While the structural component includes human resources, hospital committee, and information technology infrastructure, the process component comprises all activities from patient discharge to submission of the diagnosis and procedure codes. At least eight health care professional disciplines are involved in the coding process which comprises seven major steps, each of which involves different hospital staff: 1) Discharge Summarization, 2) Completeness Checking, 3) Diagnosis and Procedure Coding, 4) Code Checking, 5) Relative Weight Challenging, 6) Coding Report, and 7) Internal Audit. The hospital coding practice can be affected by at least five main factors: 1) Internal Dynamics, 2) Management Context, 3) Financial Dependency, 4) Resource and Capacity, and 5) External Factors.

**Conclusions:**

Hospital coding practice comprises both structural and process components, involves many health care professional disciplines, and is greatly varied across hospitals as a result of five main factors.

## Background

The Universal Coverage (UC) scheme was introduced in Thailand in 2001 to provide health benefits to approximately three quarters of the Thai citizens who are not covered by any of the health insurance and welfare schemes (i.e. children, elderly, unemployed, and workers in informal sector). The scheme is financed from general taxation and has been administered by the National Health Security Office (NHSO), under the supervision of the Public Health Minister [1]. All public hospitals are mandated to join the UC scheme while some private hospitals may voluntarily participate. Hospital providers are paid for outpatient and preventive services based on prospective capitation whereas Diagnosis Related Grouping (DRG)-based retrospective payment with a global budget-a proposed annual budget for medical expenditure under the UC scheme-is used for inpatient care.

DRG is "a system to classify hospital cases into groups, expected to have similar resource use" [2]. It was introduced in the USA and then migrated to many developed and developing countries around the world. A more detailed comparative discussion on its implementation in various countries can be found elsewhere [3]. In 1998, DRG was introduced to Thailand for resource allocation in the Low Income Card scheme (LIC)-a welfare scheme for the poor that preceded UC. DRG was then developed to be an important mechanism for provider payment for inpatient care under the UC scheme in 2002.

As DRG was designed to control costs of medical treatment, it has been favored by policymakers and insurers. Use of DRG, however, can lead to coding adjustments or manipulations, as hospitals may be more likely to report diagnosis and procedure codes that result in larger reimbursements while some data might be ignored simply because of no financial incentives. This may be acceptable if the coding reflects patients' actual conditions that may consume more hospital resources. However, it is possible that some changes in hospital coding practices do not focus on patients' needs but rather on financial incentives. This phenomenon is called DRG creep, defined as "a deliberate and systematic shift in a hospital's reported case mix in order to improve reimbursement" [4].

While the system depends on complete and accurate data, the quality of diagnosis and procedure codes submitted by the hospitals has been a major concern in Thailand [5-8] and elsewhere. In 2008, the Bureau of Claims and Medical Audit (BCMA) conducted the Summary and Coding Audit on 57,828 medical records of 931 hospitals in 75 provinces (SCAD 2008). Errors were found in 42% in the abstraction from medical records to discharge summary, of which the most common mistake (28%) was secondary diagnosis. In addition, more than half of the discharge summaries were coded incorrectly. Of these coding errors, 20% could have been corrected by a certified coder [9]. Financial penalties were enforced based on these audit results; hospitals were required to return the reimbursed money of the cases submitted with incomplete and/or inaccurate codes.

The current implementation of DRG in Thailand is based on existing hospital infrastructure and human resources whereas the coding process is assumed to be 'ideal'. That is, according to BCMA, the coding process comprises two major steps. The first step after patient discharge is when the responsible physician summarizes the diagnosis and all clinical activities into a standard form called a discharge summary. Information from both real clinical care and medical records are used to produce a complete and accurate summary that reflects the real complexity of patient conditions and clinical interventions. In the second step, the certified coder assigns appropriate ICD-10 and ICD-9-CM codes relevant to the information in the Discharge Summary. The coder may also want to go back and look at the medical record or discuss with the physician when in doubt.

However, it has been suggested that the nationwide shortage of qualified coders along with the discrepancy in hospital baseline resources have made such an ideal situation unlikely [9]. A survey of 322 hospitals in 2001 revealed that only 60% of the hospitals had qualified coders; but as many as 46% of them were considered 'part-time coders' as they had to be responsible for other jobs as well [10]. Like other health professions, most of them usually work in large hospitals in urban areas. Hospitals with no qualified coders have to send some staff to attend workshops then come back to work as part-time coders.

Given that hospitals have to adapt to the UC scheme and its coding demands, there has been limited knowledge of which health care professions are involved and how the diagnosis and procedure coding is actually done within hospital settings. The objective of this study is to detail hospital coding structure and process, and to describe the roles of key hospital staff, and other related internal dynamics in Thai hospitals that affect quality of data submitted for inpatient care reimbursement.

## Methods

### Study Design and Sample

A purposive sample was conducted of 10 hospitals in Thailand, selected using Maximum Variation Sampling technique to ensure that hospital size, location (urban/rural), and type (public/private) were represented (Table 1). A hospital is considered small, medium, or large based on number of beds using government standardized cut-points for public hospitals (<= 30, 31-120, and >120 beds) whereas arbitrary cut-points for private hospitals (<= 60, 61-150, and >150 beds) were used in order to achieve comparable distribution of the number of hospitals in each group. However, there was still no large private hospital in a rural area so this category was excluded. The selected medium-sized private hospital in a rural area refused to participate because it withdrew from the UC scheme.

**Table 1:** Characteristics of Respondents in 10 Hospitals

Hospital	Type	Size	Location	Province	# Respondents
BK	Public	Small	Urban	Nonthaburi	6

NY	Public	Small	Rural	Cholburi	4

PT	Public	Medium	Urban	Phuket	3

JT	Public	Medium	Rural	Chiang Mai	3

MHR	Public	Large	Urban	Chiang Mai	5

UTR	Public	Large	Rural	Uttaradit	6

JD	Private	Small	Urban	Samutsakon	5

RK	Private	Small	Rural	Ubonratchathani	4

RC	Private	Medium	Urban	Nakonsawan	4

MI	Private	Medium	Rural	Mukdahan	(refused)

MC	Private	Large	Urban	Chiang Mai	3

-	Private	Large	Rural	(not available)	

Note: The cut points for small, medium, and large hospital size are <= 30, 31-120, and >120 beds for public hospital and <= 60, 61-150, and >150 beds for private hospitals.

### Data Collection

The study participants comprised a total of 43 people with 3-6 staff interviewed in each of the selected 10 hospitals. A cover letter and interview guides were developed and sent to the hospital directors for permission to interview their staff. Semi-structured interviews were conducted in person. The objectives and methodology of the study were explained to the respondents before the informed consents were signed. The first round of interviews started with 3 key persons who are familiar with data coding, financial, and policy processes, as identified by the hospital director. Data coders or other responsible staff were asked to describe coding process, technical aspects, and other important concerns. Financial staff were questioned about the hospital financial situation and the extent to which it was affected by this particular reimbursement budget as compared to other sources of revenue. The hospital director or a member of the executive board was asked about recent changes in hospital policy, administration, and regulation relevant to hospital coding practice. Each interviewee was then asked to identify additional staff that might help clarify some issues to be explored in additional interviews. The interviews were tape-recorded whenever possible. The voice recordings were transcribed in full by the Thai Association for the Blind (TAB).

Insofar as key informants were only asked their opinions and judgments about hospital coding practices, and were not asked about personal information, this study was therefore determined by the Johns Hopkins Bloomberg School of Public Health Institutional Review Board as not human subjects research as defined by DHHS regulations 45 CFR 46.102, and thus did not require IRB approval. Participating hospital directors in Thailand each gave permission on behalf of individual institutions.

### Data Analysis

Each unit of interview data was assigned a code to represent respondent and hospital characteristics. After all data had been collected, the lead author initially familiarized himself with the data by listening to tapes and re-reading field notes in order to list key ideas and recurrent themes until the researcher became familiar with them in their entirety. Then, a coding scheme was developed from the identified key issues and themes. This was done by drawing on a priori issues and questions derived from the study objectives, issues raised by the respondents, as well as themes that recurred in the data. During the development, the coding scheme was discussed with research assistants who also had participated in the fieldwork. As the interviews were conducted in Thai, the research assistants also helped to refine the English codes as necessary. The coding scheme comprised 99 specific codes organized within various themes. The final version of the coding scheme was used to code all transcripts. Atlas.ti 6 software (Scientific Software Development GmbH, Berlin, Germany) was used to assist in the qualitative data analysis.

## Results

### Hospital Coding Practice: Structure

Hospital coding practice was found to have structural and process components. While the structural component includes human resources, hospital committee, and information technology infrastructure, the process component comprises all activities from patient discharge to submission of the diagnosis and procedure codes. At least eight health care professional disciplines (Medical Statistician, Nurse, Physician, Public Health Staff/Paramedics, Medical Record Staff, Information Technology Staff, Finance/Accounting Staff, and others), are involved in seven major steps of coding process (Discharge Summarization, Completeness Checking, Diagnosis and Procedure Coding, Code Checking, Relative Weight Challenging, Coding Report, and Internal Audit). In this study, medical statistician is a job position that requires undergraduate-level training (as discussed later in section 3.5) and usually is responsible for analyzing patient information. With some diagnosis and procedure coding knowledge, a medical statistician is usually anticipated to work as a hospital coder.

### Human Resources

Different approaches are used for managing human resource structure in response to DRG-based reimbursement. Hospitals of different sizes differ in terms of how they utilize their staff for the coding process.

Hospital MHR, a large public hospital in an urban area, created a deputy director position for a senior physician to supervise the coding system. Physicians in each department complete the discharge summaries before they are sent to the central coding office. The hospital has 9-10 staff working as full-time coders as well as many financial staff to deal with each of the health insurance schemes.

A smaller hospital like Hospital JT appointed a mid-level physician to check and approve all medical records and discharge summary before any code was assigned. The hospital has one certified coder and three medical statisticians, who will be certified in the near future.

In Hospital NY, a small community hospital in a rural area with a high physician turn-over rate, the quality of discharge summary was dependent on the availability and cooperation of its physicians. A ward nurse and an anesthesia nurse had been assigned to work as part-time coders and code checkers, which is not in their job description.

Hospital RK, a small private hospital in a rural area, appointed a physician to do all discharge summaries on behalf of the responsible physicians. It had one nurse and one medical statistician; neither was a certified coder but had much experience. The hospital had been hiring a part-time certified coder from nearby public hospitals because of no response from any candidate to its announced job offer.

### Hospital Committee

There are two kinds of relevant hospital committees. While almost all hospitals have a committee overseeing completeness and accuracy of medical records, some hospitals also formed a committee on summary and coding audit, specifically for coding quality and reimbursement.

### IT Infrastructure

While most of the hospitals have relied on existing IT infrastructure, a few hospitals invested in more advanced technologies in order to improve the coding process. For example, Hospital MHR scans the medical record and discharge summary into a computer immediately after patient discharge to prevent from possible document loss. In addition, its IT staff also developed special software designed to check the assigned diagnosis and procedure codes. In contrast, the coder at Hospital NY doesn't have her own computer or even an office for her part-time coding task.

### Hospital Coding Practice: Process

**Figure 1:**
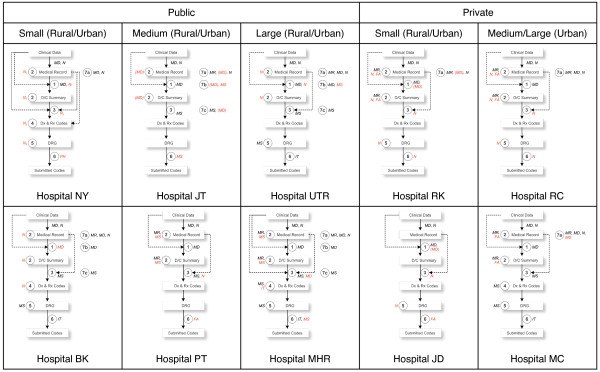
**Hospital Coding Practice of 10 Hospitals**.

As mentioned above, the Bureau of Claims and Medical Audit (BCMA)'s typical coding process comprises only two main steps: discharge summary by a responsible physician and diagnosis and procedure coding by a hospital coder. In fact, our interviews with the staff of 10 hospitals revealed that as many as seven major steps exist and a number of actors are involved in the process. The boxes and arrows in Figure 1 represent the flow of information from clinical data for patient admission to final codes submitted to BCMA. The hospital coding process starts when both physicians and nurses enter the clinical data into the medical record. Each step of the coding process is marked with the numbered circles, along with the typical actors who are responsible for it. The hospital interviews revealed some variations in hospital coding process as described below.

### Discharge Summarization

In this step, clinical data in medical records are used to fill out the discharge summary form in most hospitals. The physician responsible for the patient is considered the best person for this task as the real clinical data not recorded in the chart will implicitly be used for summarization.

Hospital NY also allows a ward nurse to help summarize when responsible physicians are not available. Hospital RK and JD appoint a physician to help the responsible physicians to do the discharge summary when necessary. Hospital MHR, the responsible physicians in some department also have to provide a code along with other relevant information on the discharge summary. In Hospital BK, each of its physicians takes weekly turns to be responsible for discharge summary of all cases.

### Completeness Checking

This step is to check the completeness of the medical record and discharge summary by staff from the medical records department. While some missing data such as hospital number and patient's demographics can be completed by the medical records staff, charts with incomplete clinical data will be returned to the ward and/or the responsible physician for correction.

In Hospital NY, a full-time ward nurse also has to work as a part-time coder. She will check the completeness of the patient chart before providing a code. In Hospital BK and UTR, ward nurses checked the medical record and discharge summary for completeness then provide comments should there be some. Hospital UTR even developed another form that contains the ward nurse's opinions on the discharge summary. This form can be used as a substitute for coding in the next step for approximately 12% of discharge summary of Hospital UTR, which are left blank by the responsible physicians after 2 weeks post-discharge. In Hospital MHR, after medical record staff checks for completeness, the chart will then be scanned for electronic storage to prevent data loss and to be used for coding.

Completeness checking is rather important for Hospital JT, a medium-sized community hospital. One of its 14 physicians is appointed to be responsible for checking all medical record and discharge summary, regardless of types of health insurance, to ensure not only completeness but also accuracy. She has to approve the discharge summary on behalf of the hospital director before the coder can give a code in the next step. If checking all records is not possible, only high-cost or long-stay cases will be checked or the hospital director will help approve those charts. Then, the responsible physician will be informed about the incomplete discharge summary.

While Hospital RK, RC, and MC also involve finance/accounting staff who will calculate the charge for inpatient care, it is only Hospital RK that attaches the calculated charge to the medical record so that the coder can compare with the discharge summary in the next step. The whole set of documents is then sent to the medical records staff to check for completeness.

### Diagnosis and Procedure Coding

This is the same as the BCMA's second step, in which the hospital coder assigns the ICD-10 code relevant to the patient's diagnosis and condition and an ICD-9-CM code relevant to clinical activities during the hospital stay. This step is expected to be done by a medical statistician who is also a certified coder. Should there be any missing or questionable information in the discharge summary or medical record, the coder will inform the responsible physician, who may or may not agree to revise.

When some hospitals do not have qualified coders (i.e. Hospital RK, NY, JD, RC), the coding will be done by some of the existing staff, especially nurses. It is therefore common to see coders who underwent training consisting of a few short courses. While private hospitals like Hospital RK, RC, and JD hire a nurse to work as a full-time coder, a ward nurse of Hospital NY has to do the coding task during her free time without additional financial incentive.

Coders in most hospitals look at both the discharge summary and medical record (i.e. Hospital MHR, RK, NY, BK, RC, MC) whereas coders in some hospitals produce codes based on the discharge summary alone (e.g. Hospital JT). Because the part-time coder of Hospital NY is also a full-time ward nurse, the real clinical condition of the patients was implicitly used in the coding process. In Hospital MHR, physicians in its family medicine department also have to do the coding as part of their training program led by a faculty physician who underwent coding training. As the physician in the previous step already approved all discharge summary and medical records, the coder of Hospital JT can rely for all their coding on the discharge summary alone.

Most respondents agree that the coder should not add codes without evidence in the discharge summary provided by the responsible physician (i.e. Hospital NY, JT, MHR, RK, UTR, RC, MC). However, coders in some hospitals seem to have more power than the physicians in the coding process. For example, the coder of Hospital PT thinks that it is acceptable to provide appropriate code as long as there is supporting evidence in the medical record. And if the responsible physician is not available, any other physician can revise the discharge summary to match the assigned code. The executive board of Hospital MHR officially allows their coders to find more codes for co-morbidity in the medical record because sometimes the physicians may have missed things and because their coders are more knowledgeable and skillful than physicians in this regard. The coders of Hospital MHR will code everything in as much detail as possible, claiming that the main purpose is to improve data quality. "If the doctor gives incomplete information on the discharge summary, we will give the code based on what we can find in the medical record," said a medical statistician of Hospital MHR. In Hospital UTR, coders are allowed to assign codes based on the nurse's version of the discharge summary if the physician version is still blank and the coding report is almost due.

Coders in some hospitals will try to find more codes from the medical record in a very systematic way (Hospital PT, RK, BK, RC); this is especially true for the procedure code. In Hospital RC, for example, the coder always looked for any procedure such as wound dressing or suturing if she found relevant information in the medical record. Likewise, the coder of Hospital RK always looked at laboratory results or the presence of a urinary catheter. "Frankly speaking, I would add more codes than what the doctors wrote in the discharge summary if I see that it cannot cover the cost of care I anticipated from the medical record I reviewed," said the coder of Hospital RK. She further explained the detailed process: "When I open the chart, I look at the admission note, doctor order, temperature form, and then laboratory result. I would add a code for anemia if the doctor prescribes folic acid or ferrous sulfate. Likewise, I would add hypokalemia or hyponatremia if the electrolyte profiles are compatible with the type of IV fluid the doctor prescribes."

### Code Checking

This step is to double check the completeness and accuracy of the assigned codes produced by hospital coder. Like the coder, the code checker should be a medical statistician who is also certified for coding knowledge. Ideally, the code checker will check the accuracy of the assigned code against information in the discharge summary.

While all cases in hospitals with small number of admission are checked, at least two methods are used by the code checker in the other hospitals to choose records to be reviewed. Code checkers of some hospitals may sample 10-30% of the discharged patient whereas those in the other hospitals may apply a criterion, either internally developed or adopted from the NHSO regional office. For example, all charts with a hospital number ending with '2' are checked.

This step is considered important for Hospital NY as seen from the fact that, despite its human resource limitation, another nurse who also underwent short course training is responsible for double-checking the assigned code. As this nurse has been trained to do basic anesthesia for occasional surgeries in this small community hospital, she has more time to be responsible for insurance-related issues including UC reimbursement and coding.

The IT staff of Hospital MHR has developed software to help the medical statisticians checking the assigned codes, focusing on some particular scenarios such as too wide range of Relative Weight, high cost, long length of stay, etc. In contrast, the ward nurse of Hospital BK checks the assigned code only for learning purposes.

### Relative Weight Challenging

This optional step focuses on exploring variations of possible Relative Weights for each of the sets of diagnosis and procedure codes. This is mostly done in the DRG grouper software provided by NHSO to see how much the Relative Weight is for each of the specified codes. It is common to see medical statisticians responsible for this task.

Although the software is anticipated to be used for learning purposes, some hospitals (Hospital NY, MHR, RK) disclosed that it actually can help maximize their revenue. "We always try all possible combinations of codes to find the maximum possible RW as long as the physicians agree," said the coder of Hospital RK. In other cases, Hospital JT for example, this step does not exist.

### Coding Report

This step is simply a submission of diagnosis and procedure coding to the National Health Security Office (NHSO). Hospitals have to send the whole database of all patients within 30 days after patient discharge. BCMA will then selectively process only the UC patients; any error found will be sent back to the hospital for correction. It is usually the hospital financial/accounting staff who receive that feedback as it means that some cases will not get reimbursed or get less than they should unless the codes are corrected. The financial/accounting staff will examine incorrect codes and forward to the medical statistician for possible correction.

Any hospital staff member who has basic computer knowledge can submit the codes. Hospital NY relies for all kinds of electronic submission on a public health professional who also is computer literate. In Hospital MHR, the medical statistician collaboratively works with IT staff to submit the codes. In Hospital RK, the coder is responsible for almost the whole coding process from medical record checking to coding report and internal audit.

### Internal Audit

As a quality control measure, this step is to ensure the quality of all relevant documentation, which includes the medical record (7a), discharge summary (7b), and diagnosis and procedure codes (7c). A Medical Record Audit uses hospital staff other than the responsible physician, most commonly a physician and/or nurse, to check completeness of the medical record using the standardized form. A medical record is considered complete if essential information such as patient's name and contact address, progress note, operation note, can be found. Medical Record Audit usually follows either the Hospital Accreditation (HA) or the Ministry of Public Health (MoPH) standard, in which the focus is not on coding but rather on overall quality of medical records.

The Discharge Summary Audit resembles BCMA's Summary Audit, which is a comparison between diagnosis and procedure in the discharge summary against information in the medical record by an auditor, given an assumption that the medical record is the gold standard. The results are assumed to represent physician performance. Because an incomplete medical record diminishes its utility as a gold standard for the Summary Audit, NHSO requires that at least 70% of medical records must be complete. Likewise, the Coding Audit is similar to BCMA's Coding Audit which is a comparison between diagnosis and procedure codes assigned by a coder against those assigned by an auditor. The result is assumed to represent coder performance, on the assumption that the auditor's coding is the gold standard.

### Factors Affecting Hospital Coding Practice

Our analysis suggested at least five major factors affecting the hospital coding practice: Internal Dynamics, Management Context, Financial Dependency, Resource & Capacity, and External Factors.

### Internal Dynamics

#### Personal drive of hospital coders

Most hospitals do not provide direct financial incentives to the coders whereas some of the incentives we saw include the opportunity to get trained outside of the hospital and some kinds of award for being a good discharge summarizer/coder. Also, most of the coders expressed their pride in helping the financial situation of the hospital, even without direct hospital incentive to do so. The coders of Hospital MHR proudly expressed their feeling for being chosen as best performers. Being a certified auditor is considered both an achievement and a source of additional income from working as a part-time auditor for BCMA.

#### Professional standard of coders

Instead of incentive, some coders expressed concern about how they have to maintain their professional standard to its highest level. The coder of Hospital JT stated that "We know how to manipulate the system to get more money but we do not do it as it violates our professional standards". Most coders are more concerned about data quality than are the hospital directors.

The head medical statistician in Hospital MHR suggested that the foundation of a medical statistician network as a new professional organization at the national level. This pioneer group would comprise only heads of medical record departments in medical school hospitals.

#### Internal conflicts

An interview with the coder of Hospital PT demonstrates a good example of how internal conflict can affect coding practice. The conflict has been so severe that at least three hospital staff, who are involved in the coding practice, decided to resign because of negative work environment from the new administration.

### Management Context

We found some variations in management context among the interviewed hospitals, which can affect how a decision to make internal changes can be made. At one extreme, Hospital NY, a 30-bed community hospital, does not have its own director. In its current setting, a senior physician at Provincial Health Office is the acting director. Unless the hospital director's signature is required, the hospital, practically speaking, has been managed by the chief nurse who has the most experience in the hospital. At the other extreme, Hospital MHR has been run by well-trained executive board members, who received at least short course management training. The internal conflicts discussed above might be because none of the executive board members of Hospital PT has received management training.

The typical management context can be represented by Hospital JT. This medium-sized community hospital has approximately 10 executive board members with no formal management training. As a public hospital, Hospital JT is mandated to participate in the UC scheme whereas private hospital participation is voluntary. All kinds of decisions have to be in compliance with government regulations on finance and human resource. For example, although the reimbursed budget is already deposited into the bank account, it cannot be legally spent until the financial department receives a letter from NHSO, which is usually delayed and no detail is provided. With a higher workload than other hospitals of similar size, staff recruitment and career pathway is still based on civil servant rules and regulations, which allow at most 2 medical statistician positions for its size. Although the hospital can find alternative channels to hire more staff, there has been no guarantee that qualified candidates will be available or interested given the current national shortage and competing offers from private sector. Hospital JT was lucky enough to be able to recruit the other two medical statisticians as public health staff whose career pathway is limited by incorrect job description and as general staff who cannot enjoy civil servant benefits.

Making a decision is not always easy within a large organization. Despite its nature of being affiliated with a medical school, Hospital MHR does not train physicians about coding due to a lack of linkage between the medical school and the hospital administration. Most of the physicians belong to the medical school but residents belong to the hospital. The hospital executive board therefore does not have 100% control over the Discharge Summary.

The difference in decision-making autonomy between public and private sectors is obvious. The executive board of Hospital MHR agrees that private hospital has more flexible decision-making process. "Private hospitals are free to compare the income against their expected cost. They can opt out if it's not profitable but we can't," said Hospital JT. When there is high workload, private hospitals can hire coders of nearby public hospitals to do coding during their free time (Hospital PT) but not vice versa. Hospital NY and RK are both small hospitals in rural area, but the private Hospital RK can easily do a proactive search for more inpatients to increase revenue. Hospital RK has relied on its family-business model with five executive board members, including the non-physician hospital director who also owns the hospital. Most of the decisions can be made in a timely manner, but still are not perfect as seen from the fact that Hospital RK failed to promptly adapt to the change in UC policy, which resulted in huge financial loss.

### Financial Dependency

Most of the selected hospitals were willing to share data about their financial information although some hospitals were concerned about being investigated later (Hospital RK). We were also surprised to hear that some private hospitals openly discuss about how they might maximize their revenue from DRG-based reimbursement. We can see that while some hospitals enjoyed a profit, others have been suffering their financial crisis (Hospital JD, JT, RK), depending on their existing financial status and how dependent their revenue is on UC inpatient care. Many hospitals are concerned about cost (Hospital MHR, JT), as compared to anticipated revenue from inpatient reimbursement. "We have a few cases who have been admitted for more than 6 months or even a year. Each of them may cost us 3-4 million [Baht] but we get only a million. And this is our loss," said a financial staff of Hospital MHR. Another staff from Hospital JT stated "We have never had enough money left to be used for any further development." "Frankly speaking, we have had no profit at all," said Hospital RK. Hospital NY has a small number of inpatients, so this source of revenue is not important while as much as one-third of Hospital JD's revenue come from UC IPD. This hospital has learned its lesson from having a too simple coding practice that depends on only one single nurse who is responsible for the whole system. The external audit conducted by BCMA revealed large discrepancies between discharge summaries and submitted codes, resulting in heavy financial penalties that affected hospital stability.

We found that it was too much of a burden and impractical to ask for a detailed financial summary from the hospitals. Moreover, we got some conflicting information from hospital executive boards and financial staff from the same hospital. A senior financial staff in Hospital MHR suggested that a simple measure like the percentage of UC IPD revenue as compared to hospital total revenue is easy to collect and can reflect the extent to which a hospital financially depends on this source of revenue. Based on this measure, the financial dependency of the hospital on this UC IPD budget varies from 7% to 65% as presented in Table 2. These numbers, it should be noted, should not be taken as official estimates, as they were based on approximations from comments made during the interviews.

**Table 2:** Financial Profiles of 8 Hospitals

Hospital	BK	NY	PT	JT	MHR	UTR	JD	RK
Type	Public	Public	Public	Public	Public	Public	Private	Private

Size	Small	Small	Medium	Medium	Large	Large	Small	Small

Location	Urban	Rural	Urban	Rural	Urban	Rural	Urban	Rural

Total Revenue*	1,200	430	1,200	3,714	81,764	21,429	100%	613

UC Revenue	686	315	-	2,000	32,486	8,572	70%	429
IPD	274	29	-	1,300	26,222	-	65%	258
OPD	412	286	-	700	6,264	-	5%	172

SC Revenue	68	86	-	-	1,817	1,071	0	0
IPD	-	-	-	-	1,428	-	0	0
OPD	-	-	-	-	389	-	0	0

CS Revenue	446	29	-	-	39,903	10,715	0	0
IPD	-	9	-	-	16,550	-	0	0
OPD	-	20	-	-	23,353	-	0	0

Other Revenue	-	-	-	-	7,558	1,071	30%	184

UC IPD/Total Revenue (%)	23%	7%	-	35%	32%	-	65%	42%

Note: All revenue amounts are approximated based on the interviews and presented in USD 1,000 (1 USD = 35 THB). If the respondent gave a range, an average was used. DRG-based Revenue = UC IPD + SC IPD + CS IPD Revenues. "-" means no data mentioned during the interviews. IPD, Inpatient Department; OPD, Outpatient Department; UC, Universal Coverage Scheme; SC, Social Security Scheme; CS, Civil Servant Medical Benefit Scheme.

Although most of the hospitals were willing to talk about all relevant issues, including their financial situation, our study still could not directly explore the DRG creep phenomenon. Respondents in most hospitals (e.g. Hospital MC, MHR, JT, NY, BK) agree that it does exist but only a few hospitals admit that they are doing it. "If you ask whether a hospital has a policy to look for the best possible codes to get the highest DRG reimbursement, I am not sure. It depends on the hospital. But we don't do it," said an executive board member and DRG coordinator of Hospital MHR. However, he agreed that financial concern is the strongest drive for hospitals to improve coding and therefore more reimbursement. "Financial concern should not exist in the coding process but it's inevitably in their blood," said the executive board member of Hospital MHR.

### Resource and Capacity

Resources available for DRG coding differ among hospitals because of nation-wide inadequate numbers and inequitable distribution of medical statisticians, especially those who are certified as coders. Hence, it is common to see 'part-time' coders from other disciplines. Their skills and knowledge unquestionably vary and depend on a number of factors. In Hospital NY, for example, the ward nurse became a part-time coder after she was sent to a few short course trainings. Over time, she has familiarized herself with major ICD categories and commonly used codes. She has made a list of diagnosis and procedure codes commonly used. She knows that a code that represents cerebral concussion is better than that of 'loss of consciousness.' Another general rules she remembers is to never use any '.9 (unspecified)' because it generates less reimbursement than a more specific code. Despite the financial importance of her work to the hospital, she has had no actual office and has to spend free time during her shift to scan through the discharge summary, find appropriate codes from the 'cheat sheet' (a self-made document containing commonly used codes) then write the assigned codes onto the discharge summary. At the other end of the spectrum, Hospital MHR has 9-10 full-time medical statisticians who work as coders, five of whom are responsible for five inpatient departments whereas the other four are responsible for outpatient tasks.

### External Factors

Hospital coding practice can be greatly affected by some external factors. First and most important is the UC policy and financing mechanism. While the policymakers want to use the DRG system to control cost for inpatient care, some hospitals expressed their negative feelings about DRG: "DRG is like the rules of the game, which should be fair, and we are just players. If you ask whether I like it, it depends on who I am. As a clinician and a medical school professor, I would say no. But if I were the policymaker, I would definitely love it," according to executive board member of Hospital MHR. When the UC policy was changed in a way that more inpatients meant more budget, Hospital RK decided to form a "patient recruitment team" to admit potential patients in the community in order to increase their revenue.

The concern about hospital coding practice also exists at the implementation level. For example, the whole coding process has to be finished within 30 days after a patient is discharged. Hospitals with inadequate staffing might not be able to meet this requirement and therefore may receive less reimbursement than they deserve, as compared to larger hospitals like Hospital MHR, which improved its medical record turnover time to reduce late code submission from 2.64% in 2007 to less than 1% in 2008.

The second factor is the BCMA audit system, which is not merely a visit by an external auditor but rather a learning network. Most hospitals agreed that the external audits could help them restrain from gaming the system. Hospitals actually formed local self-help groups and exchanged knowledge with one another to promote learning. Some experienced coders occasionally help train inexperienced ones from other hospitals. The predefined set of criteria can be modified when the audit team agrees that it is very unlikely for some small hospitals like Hospital NY to achieve. Considering human resource aspects, skillful hospital coders can be certified to be BCMA auditors and join the audit team (Hospital MHR, JT). This was also regarded as an incentive or a new career pathway for some medical statisticians. Unfortunately, certification examinations are limited to a few spots per year.

Thirdly, the production of medical statisticians has been severely inadequate. There is only one 2-year program offered by Mahidol University, which has produced approximately 50 students annually and only a few credit hours are devoted to the coding issues. The graduates can continue with the advance program for another 2 years but with no guarantee of a pay raise. The coder at Hospital PT estimated that approximately 2,000 medical statisticians have been produced but many of them have been working as either medical records or IT staff (Hospital PT, UTR). However, most respondents agree that experience is still more important than formal training (Hospital PT, UTR); having an experienced nurse as a part-time coder might be better than having a newly graduated medical statistician. Such experience is also affected by hospital setting; those who work in a small hospital will never (or only rarely) be exposed to coding complicated diseases or procedures (Hospital PT, UTR). Those who work in larger hospitals are therefore more likely to get certified than those in smaller ones.

## Discussion

**Figure 2:**
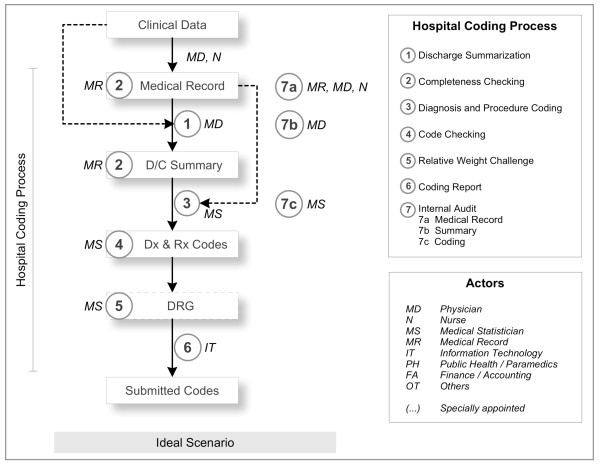
**Ideal Hospital Coding Practice**.

In this paper, we detailed the variation of coding practice in 10 selected hospitals by presenting the structural aspect and describing at least seven major steps in the hospital coding process. The coding process varies in terms of sequence of steps and type of actors involved in each step, which is affected by at least five factors we discussed earlier. We identified a more comprehensive and realistic version of the ideal hospital coding practice than that of BCMA's (Figure 2).

A number of recommendations for structural and process changes to improve data quality were revealed during our interviews (Table 3). While such changes should aim to improve overall data quality, we found that some of those changes might be differentially applied to only a specific health insurance scheme. We believe such attempts might be suggestive of hospital's intention to maximize its revenue rather than improve data quality in general.

**Table 3:** Examples of changes in Hospital Coding Practice with regard to intention to improve data quality

Data Qulity Improvement	Structure	Process
**General**	• Policy to improve quality of medical record • Form medical record audit committee • Incentive/punishment mechanisms to ensure timeliness of discharge summary completion by physician • Appoint a nurse as part-time coder • Appoint a physician to approve all discharge summary before coding • Recruit more medical statisticians • Clear career pathway for medical statisticians • Support medical statisticians to get certified as coders • Strengthen specialty-based skills of coders • Appoint a senior physician to supervise the whole coding process • Computerize medical record system	• Feedback mechanism • Revise workflow to improve medical record turnover • Weekly meeting on coding issues • Allow only physician to do the discharge summary • Physicians have to do coding themselves • Coder gives code based on discharge summary alone • Randomly select cases to check assigned code • Medical record audit results are publicly announced

**Differential**	• Form summary and coding audit committee • Have a policy to ensure physician knowledge about DRG-based reimbursement • Appoint a senior management staff to be responsible for coding practice • Have separate staff responsible for each health insurance scheme • Incentive for good discharge summarizer & coder • Contract out or use coders from outside • Appoint a staff to be responsible for DRG grouper software • Keep inpatient and outpatient records separately • Supporting tools such as cheat sheet for common codes are prepared for coder	• Check health insurance status before coding • Records of patients with different health insurance undergo different coding system • Check only codes of UC patients • Staff other than responsible physician can add/edit information in the discharge summary • Coder can offer more codes than information in discharge summary • Coder can give code if there is enough evidence in the medical record • Coder can offer codes based on laboratory results alone • Coder can add or change what the physicians wrote in the discharge summary to match anticipated cost of care • Coder can ask the responsible physician to revise diagnosis and procedure information in the discharge summary to match the code already given • Purposively select cases to check assigned code • Self-develop software to check assigned codes • DRG software is used only for UC patients • Try all possible combination of codes to find the maximum possible RW • Try to swap the principal diagnosis with the secondary diagnosis to increase RW

As financial dependency, we suggest, is the most important factor affecting hospital coding practice, we might classify hospitals based on how dependent their financial status is on this source of revenue and their data quality improvement attempts. The degree of financial dependency can be measured by our approach described in Section 3.3, whereas the extent to which the coding practice of a hospital is formalized might be measured by the number of data quality improvement activities. Ideally, we would love to see that hospitals improve data quality without concern about revenue implications, despite their financial dependency on it. But in real life we can find some other hospitals who might be in need of intervention. For example, hospitals who can survive without this budget and therefore have no interest in improving data quality, for example, or hospitals that are financially dependent on this source of revenue yet still haven't implemented data quality improvement.

The other groups of hospitals are those who have tried to improve their coding practice only because they are financially dependent on this source of revenue. While one might say that this is where DRG creep occurs, we argue that reaching such a conclusion is very difficult because coding practices are also affected by the other four uncontrollable factors (Internal Dynamics, Management Context, Resource and Capacity, and External Factors). However, the detail of their data quality improvement activities might be suggestive of their motives to game the system. It seems reasonable to suspect hospitals that involve a financial person in their coding practice or those that have differential coding practices for different health insurance schemes.

Setting the DRG creep issue aside, it is clear that coders are essential for data quality. Our findings suggest two opportunities for improvement that are worthy of NHSO's investment: producing more medical statisticians and conducting more workshops to increase the availability of part-time coders, which might be more effective and practical in the short run. The career pathway of coder in hospital setting should also be clearer.

Literature on DRG implementation has been mostly from developed countries with abundant resource or mainly about its macro-level effects. Despite difficulties in comparing DRG systems across countries [3], one common assumption is that hospital providers should be able to submit diagnosis and procedure codes with acceptable quality. We believe that our qualitative finding from a transitional country contributes to a better understanding of what actually happens within the hospital settings, especially when such assumptions might not hold.

Based on the finding from this study, a questionnaire is being developed for the national survey in our next phase, aiming to assess the national situation in order to get better idea before some policy can be formulated. The questionnaire is anticipated to be a new tool to assess hospital coding practice, which can help identify which aspect should receive external support.

## Conclusion

This qualitative study explained both structure and process components of hospital coding practice. Because of a number of factors, it argued against the assumption that hospitals are well equipped with physicians and certified coders and therefore able to submit diagnosis and procedure codes with high quality. Instead, the coding process comprises seven major steps and involves at least eight health care professional disciplines.

## Competing interests

The authors declare that they have no competing interests.

## Authors' contributions

KP conceived of and designed the study, carried out the interview, analyzed the data, and drafted the manuscript. DGW and PJW participated in its design and helped to draft the manuscript. PJW and CR helped to revise the manuscript. All authors read and approved the final manuscript.

## Pre-publication history

The pre-publication history for this paper can be accessed here:

http://www.biomedcentral.com/1472-6963/11/71/prepub
